# Predicting mortality among patients with severe COVID-19 pneumonia based on admission vital sign indices: a retrospective cohort study

**DOI:** 10.1186/s12890-023-02643-w

**Published:** 2023-09-12

**Authors:** Piyaphat Udompongpaiboon, Teeraphat Reangvilaikul, Veerapong Vattanavanit

**Affiliations:** 1 Faculty of Medicine, Prince of Songkla University, 15 Kanjanavanich Road, Hat Yai, Songkhla, 90110 Thailand; 2 Critical Care Medicine Unit, Division of Internal Medicine, Faculty of Medicine, Prince of Songkla University, 15 Kanjanavanich Road, Hat Yai, Songkhla, 90110 Thailand

**Keywords:** COVID-19, Pneumonia, Vital signs, Vital sign index, Mortality, Shock index age, CURB-65, Predictors

## Abstract

**Background:**

Coronavirus disease 2019 (COVID-19) pneumonia remains a major public health concern. Vital sign indices—shock index (SI; heart rate [HR]/systolic blood pressure [SBP]), shock index age (SIA, SI × age), MinPulse (MP; maximum HR–HR), Pulse max index (PMI; HR/maximum HR), and blood pressure–age index (BPAI; SBP/age)—are better predictors of mortality in patients with trauma compared to traditional vital signs. We hypothesized that these vital sign indices may serve as predictors of mortality in patients with severe COVID-19 pneumonia. This study aimed to describe the association between vital sign indices at admission and COVID-19 pneumonia mortality and to modify the CURB-65 with the best performing vital sign index to establish a new mortality prediction tool.

**Methods:**

This retrospective study was conducted at a tertiary care center in southern Thailand. Adult patients diagnosed with COVID-19 pneumonia were enrolled in this study between January 2020 and July 2022. Patient demographic and clinical data on admission were collected from an electronic database. The area under the receiver operating characteristic (AUC) curve analysis was used to assess the predictive power of the resultant multivariable logistic regression model after univariate and multivariate analyses of variables with identified associations with in-hospital mortality.

**Results:**

In total, 251 patients with COVID-19 pneumonia were enrolled in this study. The in-hospital mortality rate was 27.9%. Non-survivors had significantly higher HR, respiratory rate, SIA, and PMI and lower MP and BPAI than survivors. A cutoff value of 51 for SIA (AUC, 0.663; specificity, 80%) was used to predict mortality. When SIA was introduced as a modifier for the CURB-65 score, the new score (the CURSIA score) showed a higher AUC than the Acute Physiology and Chronic Health Evaluation II and CURB-65 scores (AUCs: 0.785, 0.780, and 0.774, respectively) without statistical significance.

**Conclusions:**

SIA and CURSIA scores were significantly associated with COVID-19 pneumonia mortality. These scores may contribute to better patient triage than traditional vital signs.

**Supplementary Information:**

The online version contains supplementary material available at 10.1186/s12890-023-02643-w.

## Background

The coronavirus disease 2019 (COVID-19) epidemic was quickly labeled a pandemic by the World Health Organization on March 11, 2020 [1]. Over 14.9 million deaths have been associated with the COVID-19 pandemic between January 2020 and December 2021 [2], and it has significantly threatened public health as well as the social and economic status of many countries [3].

COVID-19 can be asymptomatic or cause mild-to-severe pneumonia requiring ventilatory support and intensive care [4]. The mortality rate of severe COVID-19 pneumonia has reached 55%, which is ten times higher than that of mild cases [5]. The development of new indicators or tools that can predict the outcomes of patients with severe COVID-19 pneumonia can assist physicians in triaging and managing patients, resulting in decreased mortality [6].

Several severity scores, such as the Acute Physiology and Chronic Health Evaluation II (APACHE II) and Sequential Organ Failure Assessment (SOFA), have been used as predictors of mortality in patients with COVID-19 [7, 8]. However, these scores require laboratory values, which can be time-consuming and sometimes unavailable for rapid patient screening. The CURB-65 score is an acronym for the risk factors of Confusion, Urea, Respiratory rate, Blood pressure, and age above or below 65 years; it is a well-known simple score for triage and mortality prognosis in patients with community-acquired pneumonia [9] and severe COVID-19 [10]. The urea test is the only laboratory test required to calculate the CURB-65 score. However, CURB-age, a modification of CURB-65, is considered a better predictor than CURB-65, particularly in older patients with community-acquired pneumonia [11].

In a previous study, vital signs were obtained simply, and some of them, such as higher heart rate (HR) and respiratory rate (RR), were associated with COVID-19 mortality [12]. Vital sign indices derived from traditional vital signs, such as the shock index (SI; HR/systolic blood pressure [SBP]), shock index age (SIA; SI × age), MinPulse (MP; maximum HR–HR), Pulse max index (PMI; HR/maximum HR), and blood pressure–age index (BPAI; SBP/age), derived from traditional vital signs, are better predictors of death in patients with trauma compared to the traditional vital signs [13]. However, these vital sign indices have not been evaluated in patients with severe COVID-19 pneumonia. The primary objective was to evaluate a predictive model of mortality in patients with severe COVID-19 pneumonia, and the secondary objective was to modify the CURB-65 score using the best performing vital sign index to develop a new mortality prediction tool.

## Methods

### Study population and design

The health information system database of patients admitted to the COVID-19 ward of Songklanagarind Hospital (a university-affiliated, 800-bed, tertiary hospital in southern Thailand) between January 2020 and July 2022 was used for this retrospective cohort analysis. Patients were included if they had been admitted to the hospital and fulfilled the COVID-19 eligibility criteria for severe pneumonia. Patients were excluded if they were under 18 years old or if their vital signs recorded at the time of hospital admission were incomplete. Patients were followed up until discharge or death, whichever occurred first. The Human Ethics Committee of the Faculty of Medicine at the Prince of Songkla University approved the study protocol (EC number 65-257-14-1). The requirement for informed consent was waived due to the retrospective study design.

### Data collection

The patients’ electronic medical records were used to extract epidemiological and clinical data, such as age, sex, height, weight, comorbid conditions, number of vaccinations, vital signs at admission (including body temperature, HR, RR, and SBP, and diastolic blood pressure [DBP]), APACHE II score, CURB-65 score, requirement of mechanical ventilator support, identification of acute respiratory distress syndrome (ARDS), length of hospital stay, and in-hospital mortality.

### Definitions

COVID-19 was confirmed by a positive test result for either the rapid antigen test or the nucleic reverse transcriptase polymerase chain reaction, wherein the patient sample was obtained from a nasopharyngeal swab, throat swab, sputum, or bronchoalveolar lavage.

Severe pneumonia was defined as the presence of clinical signs of pneumonia—fever, cough, and dyspnea—and signs of severe respiratory distress, defined as accessory muscle use, inability to make full sentences while speaking due to shortness of breath, RR > 30 breaths per minute, or SpO_2_ < 90% at room air. Chest imaging modalities (radiography, computed tomography, and ultrasonography) may assist in the diagnosis, identification, and exclusion of pulmonary complications [14].

The following vital signs were measured on arrival at the COVID ward: body temperature (BT, °C), heart rate (HR, beats/min), respiratory rate (RR, breaths/min), SBP (mmHg), and DBP (mmHg). The vital sign indices were calculated based on a study by Bruijns et al. [13] The shock index (SI) was calculated by dividing HR by the SBP. SIA was calculated by multiplying the SI by age. The BPAI was calculated by dividing SBP by age. The maximum HR was calculated by subtracting the patient’s age from 220. MP was calculated by subtracting the HR from the maximum HR. PMI was calculated by dividing the HR by the maximum HR.

ARDS was diagnosed, according to the Berlin definition, as “acute onset within 1 week, bilateral lung opacities, no evidence of cardiac failure-related hydrostatic edema on echocardiography, and PaO_2_/FiO_2_ ratio < 300 mmHg with positive end-expiratory pressure ≥ 5 cm H_2_O” [15].

Illness severity was determined using APACHE II [7]. All components of the APACHE II score were recorded, and the highest and lowest values were recorded during the first 24 h of hospital admission.

The CURB-65 score comprises five parameters: confusion, RR ≥ 30 breaths per minute, blood urea nitrogen level greater than 7 mmol/L, SBP < 90 mmHg or DBP ≤ 60 mmHg, and age ≥ 65 years [9].

Mortality was defined as all-cause in-hospital mortality.

### Statistical analysis

The sample size was determined using the sample size estimate for diagnostic test research [16]. Based on the assumption that 20% of patients with severe COVID pneumonia die, we estimated that a sample size of 246 would provide 95% confidence and 80% power to detect a difference of 10% from an expected margin of error of 5% [17].

The Shapiro–Wilk test was used to assess whether the data had a normal distribution. Percentages are used to represent categorical data. For continuous data, the minimum and maximum interquartile ranges are shown together with the median values. Continuous variables and proportions were compared between the groups using the Mann–Whitney U test and chi-square test, respectively. Data for some of the inflammatory markers were missing. However, imputations for missing data were not performed in the statistical analysis.

We investigated the relationship between vital sign markers and in-hospital mortality using univariate regression analysis. In a multivariate logistic regression model with APACHE II adjustments, factors significantly associated with mortality (*P* < 0.2) in the univariate analysis were included. [18] The collinearity between variables was removed before modeling. Odds ratios (ORs) and 95% confidence intervals (CIs) were used to identify the key independent variables that influenced mortality.

For a selected set of variables, a receiver operating characteristic (ROC) curve and the area under the ROC curve (AUC) were produced. A test with an AUC of > 0.9 was considered to have high accuracy, 0.7–0.9 as moderate accuracy, 0.5–0.7 as poor accuracy, and 0.5 as chance result. [19] Based on the sensitivity, specificity, positive likelihood ratio (LR +), and negative likelihood ratio (LR -) of the vital sign indices for predicting mortality, the Youden index was developed to determine the proper cutoff values. CURB-65 was modified using vital sign indices with the best performance. To compare the differences in the AUC between the CURB-65 and modified CURB-65 scores, the Z-statistic, as reported by Hanley and McNeil, was applied [20]. Internal validation was performed using a bootstrapping resampling procedure with 1,000 replicates to evaluate the accuracy of the model. A two-tailed *P* value of < 0.05 was considered statistically significant. All statistical analyses were performed using STATA version 16 software (StataCorp, College Station, Texas, USA).

## Results

### Baseline clinical characteristics

**Fig. 1:**
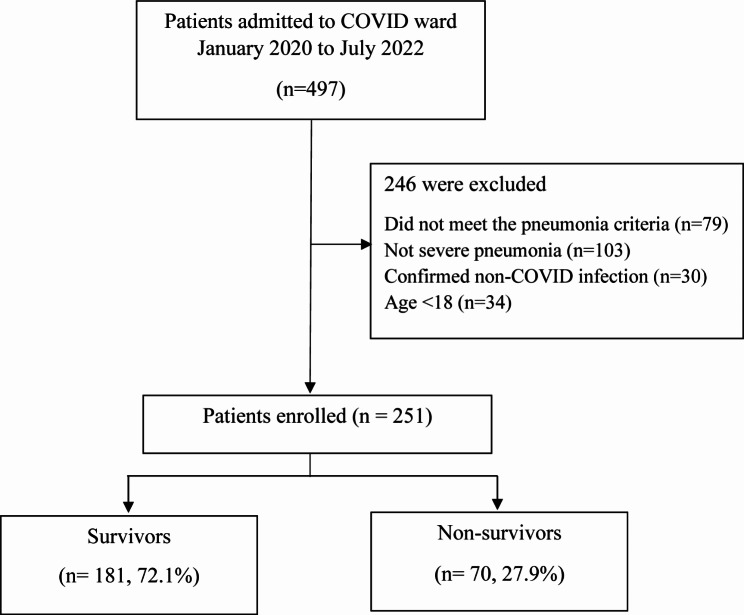
Flow diagram of the study

Of the 497 patients admitted to the COVID-19 cohort wards within the study period, 251 were enrolled in this study. **(**Fig. 1**)** The in-hospital mortality rate was 27.9%. Adult patients with COVID-19 were excluded because they did not meet the criteria for pneumonia (n = 79) or non-severe pneumonia (n = 103), and their characteristics were compared with those of patients with severe pneumonia (Table S1).

The baseline clinical characteristics of the patients with severe COVID-19 pneumonia are presented in Table 1 and Table S2. Non-survivors were older (68.5 vs. 62 years, respectively; *P* = 0.002), had lower BMI (23.2 vs. 25.3 kg/m^2^, respectively; *P* = 0.024); had a higher incidence of malignancy (7.1% vs. 0.6%, respectively; *P* = 0.027), ARDS (80% vs. 38.7%, respectively; *P* < 0.001), and chronic kidney disease (22.9% vs. 9.9%, respectively; *P* = 0.007); and had higher APACHE II scores (22 vs. 16, respectively; *P* < 0.001) compared to those survivors.

**Table 1:** Baseline characteristics of patients with severe COVID-19 pneumonia categorized by survivors and non-survivors

Characteristics	Total (n = 251)	Non–survivors (n = 70)	Survivors (n = 181)	P-value
Age, years	64 (51–76)	69 (61–81)	62 (40–75)	0.002
Sex, male	121 (48.2%)	37 (52.9%)	84 (46.4%)	0.359
BMI, kg/m^2^	24.5 (21.9–29.3)	23. 2 (21.4–27.3)	25.3 (22.1–29.7)	0.024
Comorbidities				
HTN	135 (53.8%)	41 (58.6%)	94 (51.9%)	0.344
DM	75 (29.9%)	27 (38.6%)	48 (26.5%)	0.061
Malignancy	8 (3.2%)	5 (7.1%)	3 (1.6%)	0.027
CKD	34 (13.5%)	16 (22.9%)	18 (9.9%)	0.007
CAD	16 (6.4%)	5 (7.1%)	11 (6.1%)	0.757
Stroke	24 (9.6%)	9 (12.9%)	15 (8.3%)	0.276
Chronic lung disease	24 (9.6%)	7 (10.0%)	17 (9.4%)	0.883
Vaccination	65 (25.9%)	13 (18.6%)	52 (28.7%)	0.211
Initial vital signs				
BT, °C	37.1 (36.6–37.8)	37.2 (36.4–38.0)	37.0 (36.6–37.8)	0.772
HR, beats/minute	90 (77–108)	99 (81–120)	89 (76–106)	0.018
RR, breaths/minute	24 (20–30)	26 (22–30)	24 (20–29)	0.027
SBP, mmHg	136 (119–151)	135 (118–151)	136 (120–151)	0.639
DBP, mmHg	80 (69–90)	75 (66–88)	81 (72–90)	0.078
Vital sign indices				
SI	0.67 (0.58–0.81)	0.69 (0.60–0.88)	0.66 (0.57–0.77)	0.063
SIA	41.1 (31.2–54.2)	48.7 (36.9–58.9)	38.5 (30.0–49.3)	< 0.001
MP	65.0 (42.0–82.0)	52.5 (34.8–70.3)	71.0 (48.0–85.5)	< 0.001
PMI	0.58 (0.49–0.72)	0.65 (0.52–0.79)	0.55 (0.49–0.68)	0.001
BPAI	2.13 (1.75–2.76)	1.94 (1.65–2.32)	2.31 (1.81–2.87)	0.002
APACHE II	18 (14–22)	22 (18–27)	16 (13–20)	< 0.001
CURB-65	2 (1–3)	3 (2–3)	2 (1–2)	< 0.001
Need MV support	140 (55.8)	61 (87.1)	79 (43.6)	< 0.001
Duration of MV, days	11 (4–20)	13 (8–21)	8 (4–19)	0.071
Diagnosis of ARDS	126 (50.2)	56 (80.0)	70 (38.7)	< 0.001
Ward LOS, days	15 (10–26)	15 (9–27)	15 (11–27)	0.497

Note: Data are presented as median (interquartile range) or n (%)

**Abbreviations**: APACHE II, Acute Physiology and Chronic Health Evaluation II; ARDS, acute respiratory distress syndrome; BPAI, blood pressure–age index; BMI, body mass index; BT, body temperature; CAD, coronary artery disease; CKD, chronic kidney disease; CURB-65, confusion, uremia, respiratory rate, blood pressure, age > 65 years; DBP, diastolic blood pressure; DM, diabetes mellitus; HR, heart rate; HTN, hypertension; LOS, length of stay; MP, MinPulse; MV, mechanical ventilation; PMI, pulse max index; RR, respiratory rate; SBP, systolic blood pressure; SI, shock index; SIA, shock index age

Non-survivors also had significantly higher HR (98.5 vs. 89, respectively; *P* = 0.018), RR (26 vs. 24, respectively; *P* = 0.027), SIA (48.7 vs. 38.5, respectively; *P* < 0.001), and PMI (0.65 vs. 0.55, respectively; *P* < 0.001); and significantly lower MP (52.5 vs. 71, respectively; *P* < 0.001) and BPAI (1.94 vs. 2.31, respectively; *P* = 0.002) compared to those in survivors.

### Association of vital sign indices and in-hospital mortality

The relationships between the vital sign indices and in-hospital mortality are presented in Table 2. Univariate logistic regression analysis showed that the HR, RR, SIA, MP, PMI, and BPAI were associated with mortality. Subsequently, all parameters that showed statistical significance in the univariate analysis were integrated into the multivariate logistic regression adjusted for the severity score (APACHE II); only SIA was significantly associated with in-hospital mortality.

**Table 2:** Univariate and multivariate analyses for in-hospital mortality

Variables	OR	95% CI	P-value	Adjusted OR*	95% CI	*P*-value
HR	1.02	1.01–1.03	0.005	1.01	0.99–1.02	0.274
RR	1.06	1.01–1.10	0.019	1.02	0.97–1.08	0.354
SIA	1.03	1.02–1.05	< 0.001	1.02	1.00–1.04	0.033
MP	0.98	0.96–0.99	< 0.001	0.99	1.13–1.28	0.690
PMI	35.69	5.66–225.17	< 0.001	5.35	1.13–1.29	0.100
BPAI	0.55	0.38–0.82	0.003	0.70	0.46–1.06	0.094

*Adjusted with the APACHE II score

**Abbreviations**: APACHE II, Acute Physiology and Chronic Health Evaluation II; BPAI, blood pressure-age index; CI, confidence interval; HR, heart rate; MP, MinPulse; OR, odds ratio; PMI, pulse max index; RR, respiratory rate; SI, shock index; SIA, shock index age

**Fig. 2:**
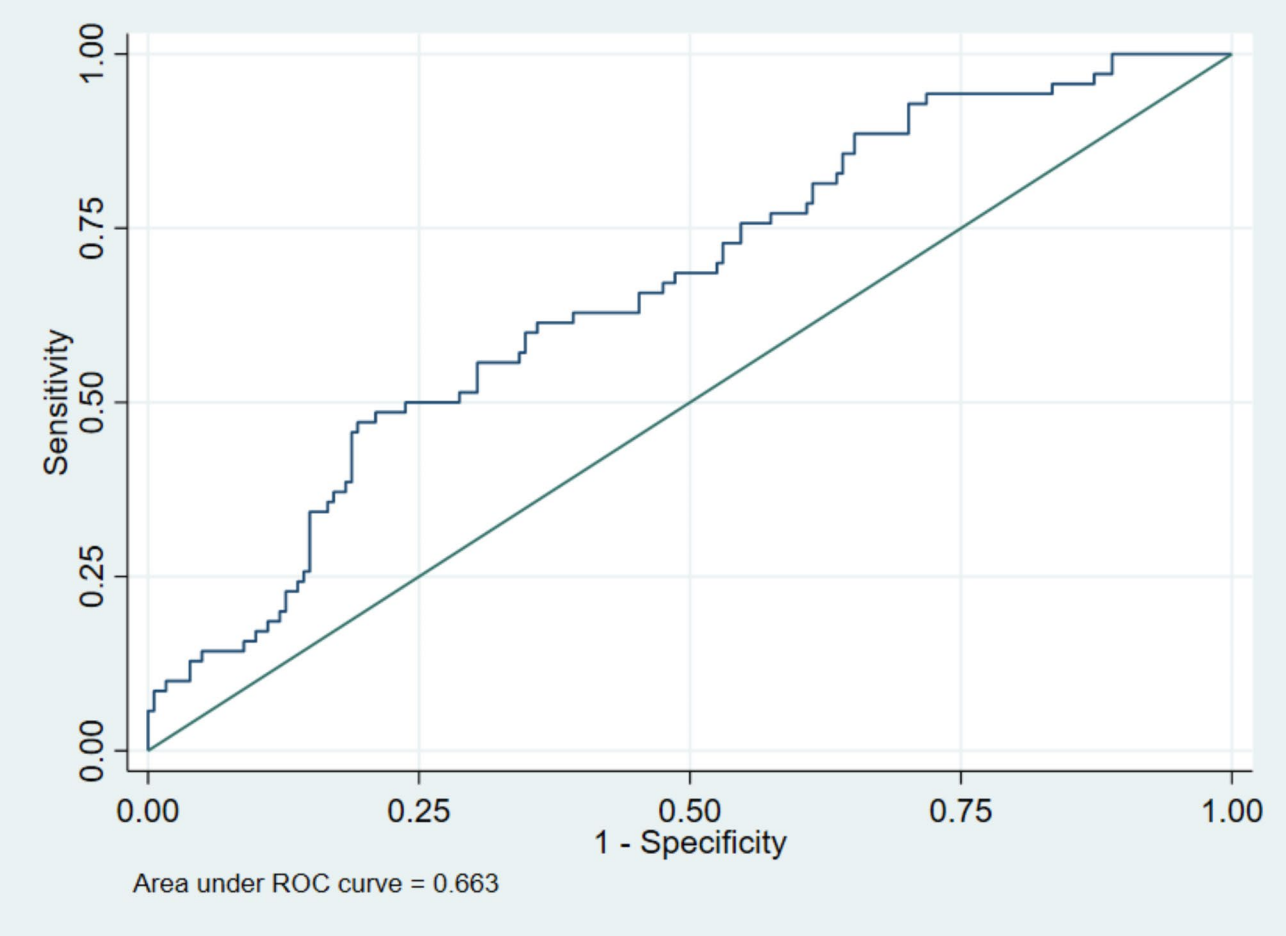
Area under the receiver operating characteristic curve for discriminating in-hospital mortality due to SIA. Abbreviations: ROC, receiver operating characteristic; SIA, shock index age

ROC curve analysis of SIA indicated an AUC of 0.663 **(**Fig. 2**).** A cutoff value of 51 for SIA provided a sensitivity of 47% and a specificity of 80% for predicting mortality in patients with COVID-19 pneumonia.

### Modified CURB-65 score proposed for evaluating mortality

**Fig. 3:**
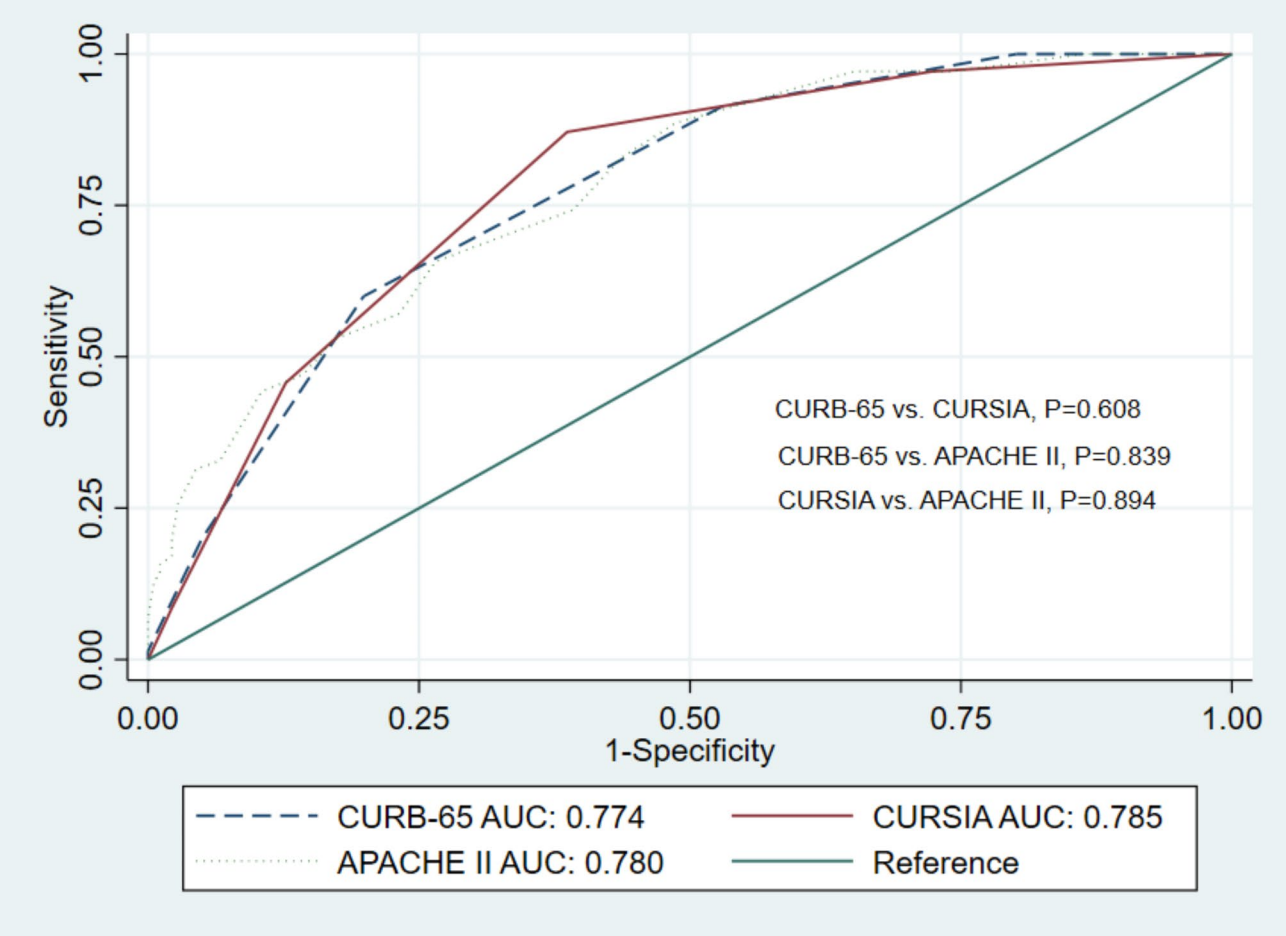
Comparison of the area under receiver operating characteristic curves of CURSIA, CURB–65, and APACHE II scores for discriminating in-hospital mortality. Abbreviations: APACHE II, Acute Physiology and Chronic Health Evaluation II; AUC, area under the receiver operating characteristic curve; CURB–65, acronym for the risk factors of Confusion, Urea, Respiratory rate, Blood pressure, and age above or below 65 years; CURSIA, modification of the CURB-65, comprising four parameters: **C**onfusion, **U**rea > 7 mmol/L, **R**R ≥ 30 breaths per minute, and **SIA** ≥ 51; SIA, shock index age

We created a new score, the CURSIA, a modification of the CURB-65, comprising four parameters: **C**onfusion, **U**rea > 7 mmol/L, **R**R ≥ 30 breaths per minute, and **SIA** ≥ 51. Each factor was assigned one point; thus, the maximum score was four. The CURSIA score showed a higher ability to predict mortality in patients with severe COVID-19 pneumonia than the APACHE II and CURB-65 scores but without statistical significance; the AUC for the CURSIA score was 0.785 (95% CI, 0.727–842), for the APACHE II score was 0.780 (95% CI, 0.720–841), and for the CURB-65 score was 0.774 (95% CI, 0.717–831). The *P* value for the pairwise comparison of the CURB-65 vs. CURSIA was 0.608, of CURB-65 vs. APACHE II was 0.839, and for the CURSIA vs. APACHE II was 0.894 **(**Fig. 3**).** We performed internal validation of the score via non-parametric ROC with 1,000 bootstrap samples, and the results showed acceptable predictive performance (AUC: 0.680, 95%CI: 0.611–0.756).

The CURSIA score with a cutoff value of ≥ 3 had a sensitivity of 45.71% and a specificity of 87.29% for predicting mortality **(**Table 3**).**

**Table 3:** Sensitivity and specificity of the CURSIA score classified by cutoff point

Cutoff point	AUC	Sensitivity	Specificity	Correctly classified	LR +	LR -
≥ 1	0.624	97.14	27.62	47.01	1.342	0.103
≥ 2	0.742	87.14	61.33	68.53	2.253	0.209
≥ 3	0.665	45.71	87.29	75.70	3.598	0.622
≥ 4	0.532	8.57	97.79	72.91	3.879	0.935

**Abbreviations**: AUC, area under the receiver operating characteristic curve; LR +, positive likelihood ratio; LR -, negative likelihood ratio

## Discussion

Herein, we aimed to describe the association between vital sign indices at admission and COVID-19 pneumonia mortality, and to modify the CURB-65 with the best performing vital sign index to establish a new mortality prediction tool. We found that SIA was independently associated with in-hospital mortality in patients with severe COVID-19 pneumonia. A cutoff SIA value of 51 predicted mortality with a specificity of 80%. Additionally, the CURSIA or SIA-modified CURB-65 scores achieved moderate accuracy as mortality predictors (AUC, 0.785).

Although the APACHE II and SOFA scores are robust mortality stratification tools for COVID-19, their clinical application is difficult as they require patients’ laboratory values, and it is often time-consuming to obtain these. In previous studies, vital signs were reported to be associated with mortality in patients with COVID-19. However, these studies included all hospitalized patients with COVID-19, including those with less severe condition than those in our study [21]. Furthermore, some of the studies were conducted in the US and reported higher RR and HR with associated mortality, but did not report definite cutoff values [12]. To our knowledge, this is the first study to report SIA as a predictor of mortality in patients with severe COVID-19 pneumonia.

SI was first described in 1967 [22] and has since been extensively researched in both emergency situations and patients with COVID-19 [21]. By contrast, older patients have a less sympathetically responsive HR and higher SBP, resulting in false-negative SI values. Therefore, Zarzaur et al. [23] introduced the SIA in 2010, which is a modification of the SI. SIA combines three components—HR, systolic blood pressure, and age—to create a singular marker and is a prognostic marker in patients with trauma and myocardial infarction. Zarzaur et al. reported that an SIA threshold greater than 39.3 was predictive of a higher risk of 48-h mortality, with a specificity of 81%. Bruijins et al. [13] revealed that SIA was the best mortality predictor among the vital sign indices. A cut-off value greater than 55 predicted 48-h mortality with a specificity of 95% and sensitivity of 42.3%. In patients with acute myocardial infarction undergoing percutaneous coronary intervention, SIA alone can identify individuals at high risk of death [24].

Based on our results, the other vital signs and vital sign indices did not correlate with mortality. There are several limitations to using the conventional body temperature, HR, and RR. Only 50% of patients who test positive for severe acute respiratory syndrome coronavirus-2 at their initial presentation have a body temperature of > 37 °C [25]. Body temperature and HR at admission did not show a significant relationship with mortality in hospitalized patients with COVID-19 [26]. However, another study reported that HR variability, analyzed using 12-lead electrocardiography, was associated with COVID-19 survival [27]. The BPAI combines two components: SBP and age. At an advanced age, SBP is affected by an inappropriate sympathetic response, resulting in false-negative values. MP and PMI use the maximum HR. The maximum HR was defined as 220 minus the patient age, and this formula could be underestimated, especially in older adults [28].

The CURB-65 is a well-known scoring system proposed by the American Thoracic Society for community-acquired pneumonia [9]. In contrast, the CURB-65 score has poorer specificity in older adults, and adding the “age of above/below 65” criterion to CURB does not increase its sensitivity or specificity in hospitalized patients [29]. CURB-65 was modified to CURB-age [11], expanded CURB-65 [30], and modified CURB-65 [31] for better prognostic performance. During the COVID-19 pandemic, CURB-65 was used as a prognostic predictor. In our study, the addition of SIA improved the AUC of CURB-65 from 0.774 to 0.785, which was higher than that of the APACHE II score; however, there was no statistically significant difference between these three scores.

Our study had some limitations. First, this was a single-center, retrospective study with a small sample size, and therefore, the findings of the study cannot be generalized. Second, we only focused on patients with severe COVID-19 pneumonia; other forms of COVID-19 pneumonia were not analyzed. Third, we did not assess medications that affect vital signs such as beta-blockers, and arrhythmias were not identified. Fourth, vital signs were measured at a single time point, which may not accurately reflect disease dynamics. The SIA and CURSIA scores should be externally validated in a large-scale prospective study before their application in clinical practice.

## Conclusions

This study demonstrated that the SIA and CURSIA scores were significantly associated with COVID-19 pneumonia mortality and may contribute to better patient triage than traditional vital signs. SIA and CURSIA can be easily retrieved and calculated, and can be applied for risk stratification of mortality in patients with severe COVID-19 pneumonia who require intensive care.

### Electronic supplementary material

Below is the link to the electronic supplementary material.

Supplementary Material 1

## Data Availability

The datasets used and/or analyzed in the current study are available from the corresponding author upon reasonable request.

## List of Abbreviations

APACHE II: Acute Physiology and Chronic Health Evaluation II
ARDS: acute respiratory distress syndrome
BPAI: blood pressure–age index
BMI: body mass index
BT: body temperature
CAD: coronary artery disease
CKD: chronic kidney disease
DBP: diastolic blood pressure
DM: diabetes mellitus
HR: heart rate
HTN: hypertension
LOS: length of stay
MP: MinPulse
MV: mechanical ventilation
PMI: pulse max index
RR: respiratory rate
SBP: systolic blood pressure
SI: shock index
SIA: shock index age
