# Supplementary material for: Predicting mortality among patients with severe COVID-19 pneumonia based on admission vital sign indices: a retrospective cohort study
Source: BMC Pulm Med. 2023 Sep 12;23:342. doi: 10.1186/s12890-023-02643-w (PMC10496301; doi:10.1186/s12890-023-02643-w)
Supplement: Supplementary file 1 — Supplementary Material 1 [file 12890_2023_2643_MOESM1_ESM.docx]

**Additional file 1**

**Table S1.** Baseline characteristics of COVID-19 patients with no pneumonia, mild pneumonia, and severe pneumonia

| **Characteristics** | **No pneumonia**  **(n =79)** | **Mild pneumonia**  **(n =103)** | **Severe pneumonia**  **(n=251)** | **P-value*** |
| --- | --- | --- | --- | --- |
| Age, years | 39 (28–55) | 44 (30-64) | 64 (51–76) | < 0.001 |
| Sex, male | 33 (41.8%) | 39 (37.9%) | 121 (48.2%) | 0.176 |
| **Initial vital signs** |  |  |  |  |
| BT, °C | 36.8 (36.4–37.5) | 36.9 (36.6–37.4) | 37.1 (36.6–37.8) | 0.015 |
| HR, beats/minute | 88 (76–96) | 87 (77–98) | 90 (77–108) | 0.010 |
| RR, breaths/minute | 20 (18–22) | 20 (19–24) | 24 (20–30) | <0.001 |
| SBP, mmHg | 127 (116–145) | 130 (118–143) | 136 (119–151) | 0.061 |
| DBP, mmHg | 80 (74–89) | 81 (72–91) | 80 (69–90) | 0.521 |
| **Vital sign indices** |  |  |  |  |
| SI | 0.69 (0.58–0.79) | 0.66 (0.58–0.76) | 0.67 (0.58–0.81) | 0.667 |
| SIA | 25.8 (19.7–32.5) | 29.9 (22.4–38.1) | 41.1 (31.2–54.2) | <0.001 |
| MP | 92.0 (76.0–104.0) | 83.0 (72.0–96.0) | 65.0 (42.0–82.0) | <0.001 |
| PMI | 0.49 (0.44-0.54) | 0.50 (0.47-0.57) | 0.58 (0.49–0.72) | <0.001 |
| BPAI | 3.33 (2.56–4.53) | 2.94 (2.18–4.11) | 2.13 (1.75–2.76) | <0.001 |
| **Severity scores** |  |  |  |  |
| APACHE II | 10 (8–14) | 10 (8–14) | 18 (14–22) | <0.001 |
| CURB-65 | 0 (0–1) | 0 (0–1) | 2 (1–3) | <0.001 |
| **Outcomes** |  |  |  |  |
| Ward LOS, days | 9 (6–12) | 12 (10–14) | 15 (10–26) | <0.001 |
| In-hospital mortality | 2 (2.5%) | 0 (0) | 70 (27.9%) | <0.001 |

Note**:** Data are presented as median (interquartile range) or n (%).

**P*-values for categorical variables were calculated using the chi-square test, and those for continuous variables were calculated using the Kruskal-Wallis test.

**Abbreviations:** APACHE II, Acute Physiology and Chronic Health Evaluation II; BPAI, blood pressure–age index; BT, body temperature; CURB-65, confusion, uremia, respiratory rate, blood pressure, age > 65 years; DBP, diastolic blood pressure; HR, heart rate; LOS, length of stay; MP, MinPulse; PMI, pulse max index; RR, respiratory rate; SBP, systolic blood pressure; SI, shock index; SIA, shock index age

**Table S2.** Other baseline characteristics of patients with severe COVID-19 pneumonia categorized as survivors and non-survivors

| **Characteristics** | **Total**  **(n=251)** | **Non-survivors**  **(n = 70)** | **Survivors**  **(n = 181)** | **P-value** |
| --- | --- | --- | --- | --- |
| **No. of COVID-19 vaccination** |  |  |  | 0.347 |
| 0 | 172 (68.5%) | 54 (77.1%) | 118 (65.2%) |  |
| 1 | 37 (14.7%) | 7 (10%) | 30 (16.6%) |  |
| 2 | 27 (10.8%) | 6 (8.6%) | 21 (11.6%) |  |
| 3 | 1 (0.4%) | 0 (0) | 1 (0.6%) |  |
| Unknown | 14 (5.6%) | 3 (4.3%) | 11 (6.1%) |  |
| **Treatment** |  |  |  |  |
| Favipiravir | 123 (49%) | 30 (42.9%) | 93 (51.4%) | 0.226 |
| Remdesivir | 213 (84.9%) | 61 (87.1%) | 152 (83.9%) | 0.530 |
| Steroid | 229 (91.2%) | 64 (91.4%) | 165 (91.2%) | 0.946 |
| Convalescent plasma | 16 (6.4%) | 4 (5.7%) | 12 (6.6%) | 0.790 |
| IL-6 inhibitor | 32 (17.8%) | 14 (20%) | 18 (9.9%) | 0.032 |
| Janus kinase inhibitor | 21 (8.4%) | 3 (4.3%) | 18 (9.9%) | 0.146 |
| Vasopressor | 96 (38.2%) | 54 (77.1%) | 42 (23.2%) | < 0.001 |
| RRT | 11 (4.4%) | 6 (8.6%) | 5 (2.8%) | 0.044 |
| Hemoperfusion | 11 (4.4%) | 6 (8.6%) | 5 (2.8%) | 0.044 |
| ECMO | 5 (1.2%) | 2 (2.9%) | 3 (1.7%) | 0.542 |
| **Initial respiratory support** |  |  |  |  |
| O_2_ cannula | 36 (14.3%) | 5 (7.1%) | 31 (17.1%) | 0.043 |
| HFNC | 115 (45.8%) | 16 (22.9%) | 99 (54.7%) | < 0.001 |
| NIV | 2 (0.8%) | 0 | 2 (1.1%) | 0.376 |
| MV | 98 (39%) | 49 (70%) | 49 (27.1%) | < 0.001 |
| **PaO_2_/FiO_2_ ratio** | 113.7 (77.5–149.2) | 98.5 (69.3–138.9) | 126.7 (82.3–174.5) | 0.023 |
| **Inflammatory markers*** |  |  |  |  |
| CRP, mg | 56.7 (21.6–109.2) | 74.4 (32.9–153.2) | 51.3 (20–94.4) | 0.012 |
| Ferritin, mcg/L | 946.5 (350–1836.5) | 1229 (721.5–2000) | 854.5 (291–1517) | 0.006 |
| IL-6, pg/mL | 50.5 (18.3–110) | 120.5 (52.3–349) | 34.9 (13.2–67.1) | < 0.001 |
| **Nosocomial infection** |  |  |  |  |
| VAP/HAP | 69 (27.5%) | 39 (55.7%) | 30 (16.6%) | < 0.001 |
| BSI | 23 (9.2%) | 11 (15.7%) | 12 (6.6%) | 0.025 |
| CRBSI | 6 (2.4%) | 4 (5.7%) | 2 (1.1%) | 0.032 |
| UTI | 49 (19.5%) | 15 (21.4%) | 34 (18.8%) | 0.743 |

Note**:** Data are presented as median (interquartile range) or n (%).

* Missing data were identified for CRP (n=4), ferritin (n=43), and IL-6 (n=4) levels.

**Abbreviations:** COVID-19, coronavirus disease 2019; BSI, bloodstream infection; CRBSI, catheter-related bloodstream infection; CRP, C-reactive protein; ECMO, extracorporeal membrane oxygenation; HAP, hospital-acquired pneumonia; HFNC, high-flow nasal cannula; IL-6, interleukin 6; NIV, noninvasive ventilation; MV, mechanical ventilation; RRT, renal replacement therapy; UTI, urinary tract infection; VAP, ventilator-associated pneumonia.
